# The Action of 2-Aminoethyldiphenyl Borinate on the Pulmonary Arterial Hypertension and Remodeling of High-Altitude Hypoxemic Lambs

**DOI:** 10.3389/fphys.2021.765281

**Published:** 2022-01-10

**Authors:** Sebastián Castillo-Galán, Daniela Parrau, Ismael Hernández, Sebastián Quezada, Marcela Díaz, Germán Ebensperger, Emilio A. Herrera, Fernando A. Moraga, Rodrigo Iturriaga, Aníbal J. Llanos, Roberto V. Reyes

**Affiliations:** ^1^Laboratorio de Neurobiología, Facultad de Ciencias Biológicas, Pontificia Universidad Católica de Chile, Santiago, Chile; ^2^Unidad de Fisiología y Fisiopatología Perinatal, Programa de Fisiopatología, Facultad de Medicina, Instituto de Ciencias Biomédicas, Universidad de Chile, Santiago, Chile; ^3^School of Health and Biomedical Sciences, RMIT University, Bundoora, VIC, Australia; ^4^Departamento de Promoción de la Salud de la Mujer y el Recién Nacido, Facultad de Medicina, Universidad de Chile, Santiago, Chile; ^5^International Center for Andean Studies, Universidad de Chile, Santiago, Chile; ^6^Laboratorio de Fisiología, Hipoxia y Función Vascular, Departamento de Ciencias Biomédicas, Facultad de Medicina, Universidad Católica del Norte, Coquimbo, Chile; ^7^Centro de Excelencia en Biomedicina de Magallanes, Universidad de Magallanes, Punta Arenas, Chile

**Keywords:** neonatal pulmonary hypertension, hypoxia, high altitude, 2-APB, store operated channels

## Abstract

Calcium signaling is key for the contraction, differentiation, and proliferation of pulmonary arterial smooth muscle cells. Furthermore, calcium influx through store-operated channels (SOCs) is particularly important in the vasoconstrictor response to hypoxia. Previously, we found a decrease in pulmonary hypertension and remodeling in normoxic newborn lambs partially gestated under chronic hypoxia, when treated with 2-aminoethyldiphenyl borinate (2-APB), a non-specific SOC blocker. However, the effects of 2-APB are unknown in neonates completely gestated, born, and raised under environmental hypoxia. Accordingly, we studied the effects of 2-APB-treatment on the cardiopulmonary variables in lambs under chronic hypobaric hypoxia. Experiments were done in nine newborn lambs gestated, born, and raised in high altitude (3,600 m): five animals were treated with 2-APB [intravenous (i.v.) 10 mg kg^–1^] for 10 days, while other four animals received vehicle. During the treatment, cardiopulmonary variables were measured daily, and these were also evaluated during an acute episode of superimposed hypoxia, 1 day after the end of the treatment. Furthermore, pulmonary vascular remodeling was assessed by histological analysis 2 days after the end of the treatment. Basal cardiac output and mean systemic arterial pressure (SAP) and resistance from 2-APB- and vehicle-treated lambs did not differ along with the treatment. Mean pulmonary arterial pressure (mPAP) decreased after the first day of 2-APB treatment and remained lower than the vehicle-treated group until the third day, and during the fifth, sixth, and ninth day of treatment. The net mPAP increase in response to acute hypoxia did not change, but the pressure area under the curve (AUC) during hypoxia was slightly lower in 2-APB-treated lambs than in vehicle-treated lambs. Moreover, the 2-APB treatment decreased the pulmonary arterial wall thickness and the α-actin immunoreactivity and increased the luminal area with no changes in the vascular density. Our findings show that 2-APB treatment partially reduced the contractile hypoxic response and reverted the pulmonary vascular remodeling, but this is not enough to normalize the pulmonary hemodynamics in chronically hypoxic newborn lambs.

## Introduction

The failure in the fetal-to-neonatal transition ensuing in a functional pulmonary circulation after birth results in pulmonary hypertension of the newborn, a syndrome that affects up to 6.8 per 1,000 of the births in lowlands (Walsh-Sukys et al., 2000), but whose prevalence could reach ∼10% of neonates under hypobaric hypoxia of the Andean Altiplano (Keyes et al., 2003; Peñaloza and Arias-Stella, 2007; Herrera et al., 2015). The pulmonary hypertension of the newborn is characterized by high postnatal mean pulmonary arterial pressure (mPAP), pulmonary vascular resistance (PVR), and contractility, resulting from an imbalance between vasoconstrictor and vasodilator mechanisms, and a pathological remodeling involving hyperplasic/hypertrophic growth of the wall of distal pulmonary arteries (Gao and Raj, 2011; Suresh and Shimoda, 2016; Mathew and Lakshminrusimha, 2017). The persistent high mPAP can lead to right ventricle hypertrophy, cardiac failure, and death. Moreover, survivors may have impaired neurological development and recurrent pulmonary hypertension later in life (Nair and Lakshminrusimha, 2014). Both fetal and postnatal pulmonary arteries quickly contract and develop a pathological remodeling in response to hypoxia (Gao and Raj, 2011; Dunham-Snary et al., 2017; Hussain et al., 2017). Therefore, the high-altitude chronic hypoxia during pregnancy and early after birth results in pulmonary hypertension of the newborn and associated cardiopulmonary risk later in life (Herrera et al., 2015; Gonzaléz-Candia et al., 2020). We have previously established two ovine models of neonatal pulmonary hypertension induced by high-altitude hypoxia. In the first model, conception, 100% of gestation, birth, and postnatal study were conducted at 3,600 meters above sea level (m.a.s.l) (Herrera et al., 2007). In the second model, conception and first 30% of gestation occurred at lowland; the last 70% of gestation and birth at 3,600 m.a.s.l. to return to the lowland at 2 days of postnatal age for the study (Herrera et al., 2010). Both models of perinatal hypoxia exposure showed increased mPAP, PVR, and hypoxic pulmonary vasoconstriction, right ventricular hypertrophy, and thickened pulmonary arterial walls compared with lowland healthy controls. Nevertheless, the first model is hypoxemic (Herrera et al., 2007), while the second is normoxemic after birth, simulating in the latter, the human neonatal pulmonary hypertension that persists despite oxygenation (Herrera et al., 2010). Store-operated Ca^2+^ entry (SOCE) is a Ca^2+^ influx across the plasma membrane, which is activated by a decrease in intraluminal Ca^2+^ of sarcoplasmic reticulum released through inositol triphosphate receptors (IP_3_R) or ryanodine receptors. SOCE allows replenishment of Ca^2+^ stores, but it can itself generate long-term Ca^2+^ signals. The major components of store-operated channels (SOCs) are Orai1 and stromal interacting molecule 1 (Stim1) that form the pore at the plasma membrane and the Ca^2+^ sensor of the sarcoplasmic reticulum, respectively. In resting cells, Stim1 is uniformly found in the sarcoplasmic reticulum. Fall of Ca^2+^ from sarcoplasmic reticulum results in oligomerization and redistribution of Stim1 near the plasma membrane to activate Orai1 and initiate an inwardly rectifying and Ca^2+^-selective current known as Ca^2+^-release-activated Ca^2+^ current (Icrac). In both pulmonary artery smooth muscle and endothelial cells, this Ca^2+^ entry through Orai1 allows recruitment, and activation of transient receptor potential canonical (TRPC), mainly TRPC1 to provide additional and less selective Ca^2+^ influx. The sum of both Orai- and TRPC-mediated currents generates a sustained Ca^2+^ influx termed store-operated Ca^2+^ current (Isoc) (Fernandez et al., 2012; Earley and Brayden, 2015; Ambudkar et al., 2017; Putney, 2018; Reyes et al., 2018a). There is great evidence about the involvement of this Ca^2+^ signaling complex in adults forms hypoxic pulmonary over-constriction and remodeling, the main components of pulmonary hypertension (Reyes et al., 2018a). Our group has also found evidence on the participation of these channels on neonatal pulmonary hypertension in ovine models. Thus, the treatment of normoxic pulmonary hypertensive newborn lambs with 2-aminoethyldiphenyl borinate (2-APB), a non-selective inhibitor of store-operated Ca^2+^ signaling, results in a partial reversal of pulmonary hypertension (Castillo-Galán et al., 2016). In contrast, we have observed that these channels are functionally upregulated in the pulmonary circulation of hypoxic high-altitude newborn lambs where they contribute to increased vasoconstrictor response to acute hypoxia (Parrau et al., 2013). Nevertheless, in addition to their hypoxemia, highland lambs also differ from lambs with partial gestation at high altitude in their greater pulmonary arterial medial layer thickening (Quezada et al., 2014). The window of perinatal hypoxia exposure also determines differences in key proteins involved in vascular contraction and remodeling: soluble guanylate cyclase (sGC) is downregulated in the high-altitude lambs but is unchanged in lambs with partial gestation at highlands, while big conductance Ca^2+^-dependent potassium channel (BKCa channels) expression is constant in the former and upregulated in the later (Herrera et al., 2008a,^2010^, ^2019^). These differences could have a great influence on the response to treatments against pulmonary hypertension. The ability to reverse pulmonary hypertension has not been studied in lambs with gestation, birth, and postnatal development in high altitude. In this study, we evaluated the effect of treatment with 2-APB in hemodynamic variables and pulmonary vascular remodeling of lambs developed and raised at 3,600 m.a.s.l.

## Materials and Methods

All experimental protocols were reviewed and approved by the Faculty of Medicine Bioethics Committee of the University of Chile (CBA 0476 and 1172 FMUCH). Animal care, maintenance, procedures, and experimentation were performed in accordance with the Guide for the Care and Use of Laboratory Animals published by the National Institutes of Health (NIH Publication No. 85-23, Revised 1996) and adhered to the ARRIVE guidelines.

### Animals

Experiments were performed on 10 newborn sheep (*Ovis aries*) gestated, born, and raised at Putre Research Station, International Center for Andean Studies (INCAS), at 3,600 m.a.s.l.

### Surgical Preparation and *in vivo* Experiments

At 3 days old, newborn lambs were chronically instrumented for *in vivo* studies as reported previously (Herrera et al., 2007, 2010; Parrau et al., 2013; Castillo-Galán et al., 2016). Briefly, animals were anesthetized with a combination of ketamine [10 mg/kg, intramuscularly (i.m.)] and xylazine (0.05 mg/kg i.m.) and also an additional local infiltration of 2% lidocaine. Polyvinyl catheters were placed into the femoral artery to record systemic variables and a Swan–Ganz catheter was placed into the pulmonary artery to record cardiopulmonary variables. Oxytetracycline [20 mg/kg, subcutaneously (s.c.)] and sodium metamizole (0.1 mg/kg i.m.) were given for 3 days after surgery (Herrera et al., 2007, 2010; Parrau et al., 2013; Castillo-Galán et al., 2016).

At 4 days old, the lambs were randomly divided into two groups and submitted to 10-day treatment with a single daily dose of infusion vehicle [dimethyl sulfoxide (DMSO): NaCl 1:10 intravenous (i.v.), *n* = 4] or 2-APB infusion (10 mg/kg/day bolus in vehicle i.v., *n* = 5) every morning. A similar dose of 2-APB administered to lambs with partial gestation under chronic hypoxia induced marked hemodynamic improvement (Castillo-Galán et al., 2016). Pulmonary arterial pressure (PAP), systemic arterial pressure (SAP), and heart rate (HR) were recorded *via* a data acquisition system (PowerLab/8SP System, ADInstruments, Bella Vista, NSW, Australia and Lab Chart version 7.0 Software, ADInstruments, Castle Hill, NSW, Australia) connected to a computer. Cardiac output (CO) was determined using the thermodilution method by injecting 3 ml of chilled (0°C) 0.9% NaCl into the pulmonary artery through the Swan–Ganz catheter connected to a CO computer (COM-2, Baxter, Healthcare Corporation’s Edwards Critical-Care Division, United States). mPAP, mean SAP (mSAP), PVR, and systemic vascular resistance (SVR) were calculated as described previously (Herrera et al., 2007, 2010, 2019; Parrau et al., 2013; Castillo-Galán et al., 2016). Arterial blood samples were taken daily to determine arterial pH, PO_2_, PCO_2_, hemoglobin saturation percentage (SaO_2_), total hemoglobin (THb), and oxygen content (O_2_ct) (IL-synthesis 25, Instrumentation Laboratories, AACC’s Washington DC, United States; measurements corrected to 39°C). Cardiopulmonary variables and arterial blood gasses were measured every day, 5 min before the infusion, so that the first day corresponds to the basal condition before starting the treatment, and on the following days, values were recorded 24 h after the infusion of the previous day.

Twenty-four hours after the last infusion, animals were subjected to a 3-h experimental protocol of a superimposed acute hypoxic challenge as previously described (Herrera et al., 2010, 2019; Parrau et al., 2013; Castillo-Galán et al., 2016), consisting in 1 h of basal recording (breathing room air), 1 h of superimposed isocapnic hypoxia, and 1 h of recovery during which they returned to breath room air. Superimposed isocapnic hypoxia was induced *via* a transparent, loosely tied polyethylene bag placed over the head of the animal into which a known mixture of air, N_2_, and CO_2_ was passed at a rate of 20 L/min to reach an arterial PO_2_ of ∼30 mmHg with a constant PCO_2_. Cardiopulmonary variables and arterial blood gasses were also recorded during this protocol. The contractile response of the pulmonary circulation to acute hypoxia after the end of the treatment was calculated as the mPAP/ΔPO_2_ ratio or the area under the curve (AUC) as described previously (Castillo-Galán et al., 2016; Reyes et al., 2018b).

### Euthanasia and Tissue Sampling

Twenty-four hours after the superimposed hypoxic challenge test, the lambs underwent euthanasia with an overdose of sodium thiopental (100 mg/kg i.v.), and lung samples were immediately obtained for *in vitro* analyses.

### Histological Staining and Immunohistochemistry

Lungs were perfused at 25 mmHg through the pulmonary artery with saline for blood removal, and then the left lung was perfused-fixed in 4% paraformaldehyde (PFA) in PBS 1×. Then, ∼1 cm^3^ piece of pulmonary tissue was taken from the distal portion of the central lobe, close to the pleura, and immersed-fixed with 4% PFA for 24 h at 4°C, followed by conservation in sodium azide 0.01% in 1× PBS at 4°C. The fixed tissue was embedded in paraffin, cut in 4–5 μm serial slices.

A van Gieson staining was used to assess vascular morphometry. Images were captured at 10× and 40× with an optical microscope (Olympus BX-41, Shinjuku-ku, Tokyo, Japan) coupled with a digital camera and computer. Notably, 20–25 representative resistance pulmonary arteries (150–250 μm of internal diameter) from each animal were selected for these analyses. Vascular density (number of arteries/area), percentage luminal area (luminal area/total area), and the wall, media, and adventitia thicknesses were calculated as described previously (Herrera et al., 2008b; Torres et al., 2015; Castillo-Galán et al., 2016; Castillo-Galan et al., 2020). The analysis of the microphotographs was performed with the Image Pro-Plus 6.2 software (Media Cybernetics, Inc., Rockville, MD, United States).

The immunohistochemical detection for SM-α-actin was performed with commercial antibodies (Sigma-Aldrich, Saint Louis, MO, United States, dilution: 1:400). The antigen retrieval was performed at 100°C with citrate buffer pH: 6, 1× (Dako, CA, United States). Primary antibody incubation was performed for 4 h for SM-α-actin at 4°C and labeling was developed using an HRP-diaminobenzidine kit (Envision TM+, Dako, CA, United States). The immunoreactivity of the small arteries muscle layer was calculated as described previously (Herrera et al., 2014).

### Statistical Analysis

Data are expressed as mean ± SEM. The cardiovascular variables from the baseline time point (B), from the 2-APB- and vehicle-treated lambs, were analyzed using an unpaired *t*-test and the time course using two-way ANOVA for repeated measures and the *post hoc* Newman–Keuls test. The latter test was used to analyze the cardiovascular variables from the two groups in the systemic and pulmonary responses to a superimposed hypoxic challenge. The histological and immunohistochemistry data, from the 2-APB- and vehicle-treated lambs, were analyzed using an unpaired *t*-test. For all comparisons, differences were considered statistically significant when *P* < 0.05.

## Results

### Animal Weight

The weight of the lambs used in the study was 4.90 ± 0.54–5.82 ± 0.67 kg for animals assigned to the vehicle-treated group and 4.85 ± 0.14–6.73 ± 0.29 kg for those assigned to the 2-APB-treated group at the beginning and the end of the study, respectively.

### Effect of 2-Aminoethyldiphenyl Borinate Administration on Basal Systemic and Pulmonary Variables

**FIGURE 1:**
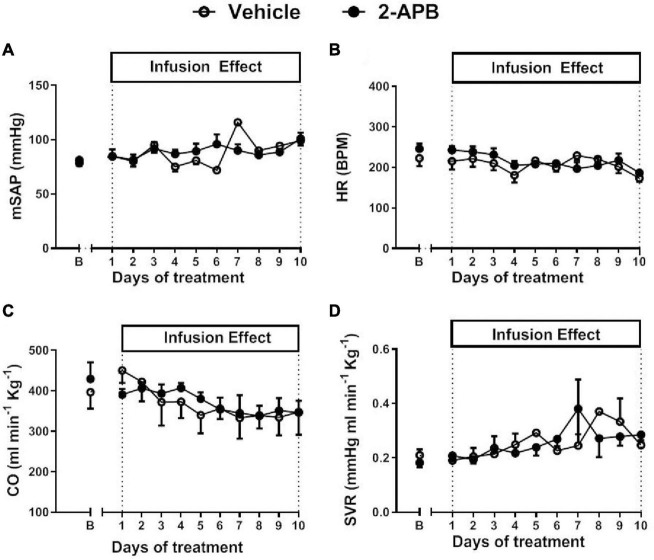
Systemic cardiovascular variables during the experimental period. Basal values for mean systemic artery pressure (mSAP) **(A)**, heart rate (HR) **(B)**, cardiac output (CO) **(C)**, and systemic vascular resistance (SVR) **(D)** were recorded 5 min before the daily infusion of vehicle (white circles) or 2-APB (black circles). The B day corresponds to basal values before treatment and the following days to values recorded 24 h after the infusion of the previous day. Values are the mean ± SEM. Statistical analysis, unpaired *t*-test for basal values, two-way ANOVA for repeated measures with Newman–Keuls *post hoc* test, for daily treatment.

**FIGURE 2:**
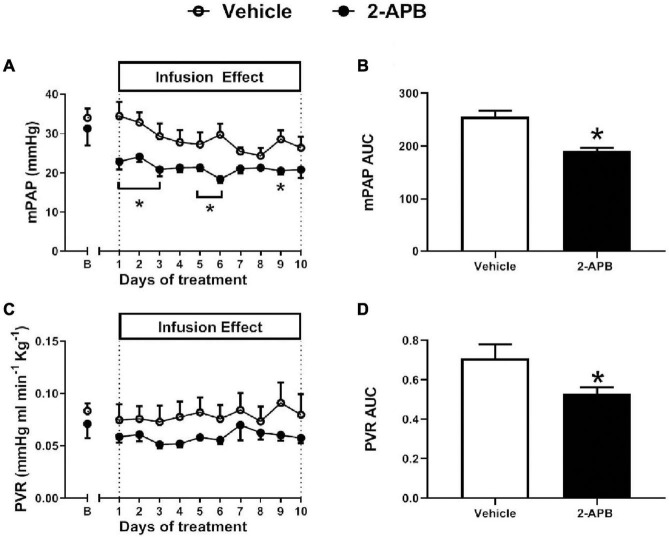
Pulmonary variables during the experimental period. Basal values for mean pulmonary artery pressure (mPAP) **(A)**, the area under the curve for mPAP (mPAP AUC) **(B)**, pulmonary vascular resistance (PVR) **(C)**, and the AUC for PVR (PVR AUC) **(D)** were measured 5 min before the daily infusion of vehicle (white symbols) or 2-APB (black symbols). The B day corresponds to basal values before starting the treatment and the following days to values recorded 24 h after the infusion of the previous day. Values are the mean ± SEM. *P ≤ 0.05, significant differences for vehicle vs. 2-APB, two-way ANOVA for repeated-measures with Newman–Keuls *post hoc* test for daily recording, unpaired *t*-test for basal values, and AUC.

The mSAP, HR, CO, and SVR were similar in control lambs and 2-APB-treated lambs along with the treatment (Figure 1). Before starting the infusion, the basal mPAP was similar in both animals assigned to the vehicle-treated group (34.04 ± 2.34 mmHg) and the 2-APB group (31.33 ± 8.55 mm Hg) (Figure 2A). However, after the first day of treatment, mPAP significantly decreased and remained lower in the 2-APB group than in the vehicle-treated group until the fourth day, and during the eighth and ninth day of treatment (Figure 2A). On the last day of treatment, the values of mPAP were similar in both groups (26.40 ± 2.83 vs. 20.85 ± 2.11 mmHg, for vehicle- and 2-APB-treated lambs, respectively) (Figure 2A). The daily recording of PVR showed similar values for both groups along the experimental period (Figure 2C). Nevertheless, the AUC along with the treatment for both mPAP and PVR, reflecting the integrated value for these variables, was lower in the 2-APB-treated lambs than in the vehicle-treated lambs (Figures 2B,D).

### Cardiovascular Response to a Superimposed Hypoxic Challenge After the Treatment

Basal values for arterial pH, PO_2_, PCO_2_, THb, SaO_2_, and O_2_ct at the end of treatment were similar in both experimental groups. During the superimposed hypoxic challenge, PO_2_, SaO_2_, and O_2_ct decreased to a similar degree reaching similar values in both experimental groups. During recovery, all these variables returned to basal values in both groups (Table 1).

**TABLE 1:** Arterial pH and blood gasses during a superimposed hypoxic challenge.

	Basal	Hypoxia	Recovery
pH			
Vehicle	7.483 ± 0.011	7.457 ± 0.014	7.457 ± 0.014
2-APB	7.520 ± 0.020	7.497 ± 0.009	7.497 ± 0.011
PO_2_, mmHg			
Vehicle	40.7 ± 2.3	30.0 ± 0.4^†^	39.9 ± 2.9
2-APB	42.4 ± 2.1	30.1 ± 0.4^†^	44.5 ± 2.5
PCO_2_, mmHg			
Vehicle	32.7 ± 1.2	33.0 ± 1.5	31.5 ± 1.5
2-APB	31.8 ± 1.7	31.8 ± 1.3	30.8 ± 1.6
S_O2_, %			
Vehicle	73.4 ± 3.5	52.9 ± 3.3^†^	70.3 ± 4.5
2-APB	70.5 ± 3.2	47.6 ± 2.5^†^	72.9 ± 3.5
THb, g/dl			
Vehicle	11.6 ± 1.1	12.1 ± 1.1	11.5 ± 1.1
2-APB	11.9 ± 0.5	12.4 ± 0.5	11.9 ± 0.6
O_2_ct, ml O_2_/dl			
Vehicle	11.0 ± 0.8	8.6 ± 0.9^†^	10.7 ± 0.8
2-APB	10.9 ± 0.3	7.9 ± 0.4^†^	11.2 ± 0.4

*Values are the mean ± SEM for arterial pH, PO_2_, PCO_2_, hemoglobin saturation (S_O2_), total hemoglobin (THb), and oxygen content (O_2_ct). 2-APB, 2-aminoethyldiphenyl borinate.*

*^†^P < 0.05, significant differences vs. all in the same group, two-way ANOVA for repeated measures with Newman–Keuls post hoc test.*

**FIGURE 3:**
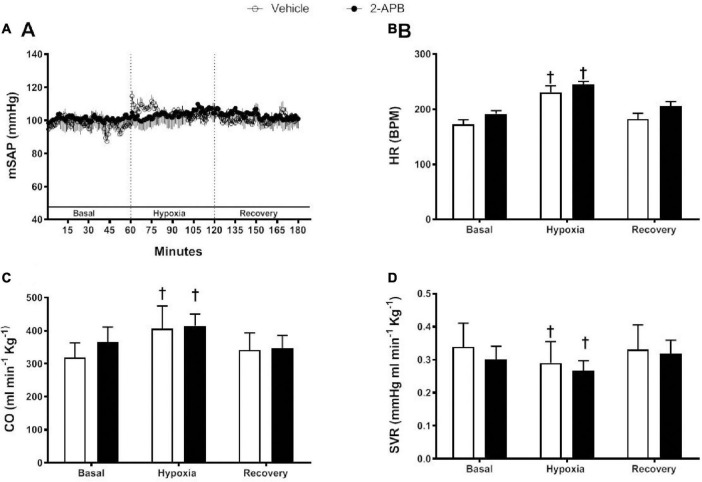
Systemic cardiovascular responses to a superimposed hypoxic challenge. Vehicle-treated (white symbols) and 2-APB-treated (black symbols) lambs were submitted to a superimposed acute hypoxic challenge 1 day after the end of the treatment and mSAP **(A)**, HR **(B)**, CO **(C)**, and SVR **(D)** were evaluated and expressed as the mean ± SEM for each experimental period. ^†^*P* ≤ 0.05, significant differences vs. all in the same group, two-way ANOVA for repeated measures with Newman–Keuls *post hoc* test.

In both experimental groups, mSAP was similar and remained stable along with the protocol (Figure 3A). HR and CO were similar in the control and 2-APB-treated lambs under basal conditions, and they showed a similar increase in both groups during acute hypoxia, and during recovery, they returned to baseline values (Figures 3B,C). The basal SVR was also similar in control and 2-APB-treated lambs decreased to similar values under acute hypoxic challenge and normalized during recovery (Figure 3D).

**FIGURE 4:**
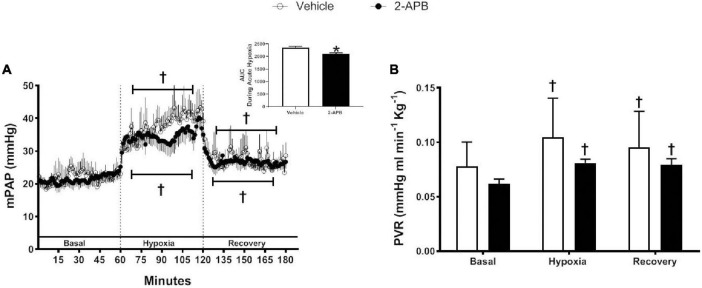
Pulmonary responses to a superimposed hypoxic challenge. Vehicle-treated (white symbols) and 2-APB-treated (black symbols) animals were submitted to a superimposed acute hypoxic challenge 1 day after the end of the treatment and mPAP, PVR, and AUC during acute hypoxia were determined as indicated in section “Materials and Methods”. **(A)** Continuous recording of mPAP, (**A**, insert) AUC during acute hypoxia, and **(B)** PVR expressed as the mean ± SEM for each experimental period. *P ≤ 0.05, significant differences for vehicle vs. 2-APB, unpaired *t*-test for AUC; ^†^*P* ≤ 0.05, significant differences vs. all in the same group, two-way ANOVA for repeated measures with Newman–Keuls *post hoc* test for acute hypoxia challenge test.

Basal mPAP was similar in control and 2-APB-treated lambs, increased during the hypoxemic challenge in both experimental groups, and decreased during recovery but remained slightly higher than the basal values (Figure 4A). The ΔmPAP/ΔPO_2_ ratio did not change with the treatment (1.26 ± 0.22 vs. 1.25 ± 0.17, for vehicle- and 2-APB-treated group, respectively), but the AUC for mPAP during the hypoxic challenge was lower in the 2-APB-treated lambs (Figure 4A insert). PVR followed a similar pattern in both vehicle and 2-APB-treated groups: basal values were similar in the control and 2-APB treated groups and increased during the acute hypoxemic challenge but did not return to baseline values during recovery (Figure 4B).

### Pulmonary Vascular Morphometry

**FIGURE 5:**
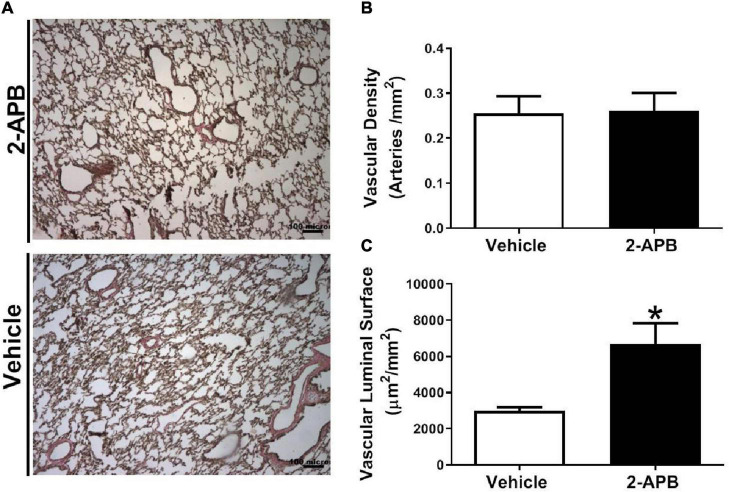
Pulmonary vascular density and luminal surface. Representative micrographs of van Gieson-stained lung sections were performed in lung slices 48 h after the end of the treatment **(A)**, vascular density **(B)**, and luminal surface area **(C)**. Scale bar = 100 μm; magnification: 10×. Values are the mean ± SEM. *P ≤ 0.05, significant differences for vehicle vs. 2-APB, unpaired *t*-test.

**FIGURE 6:**
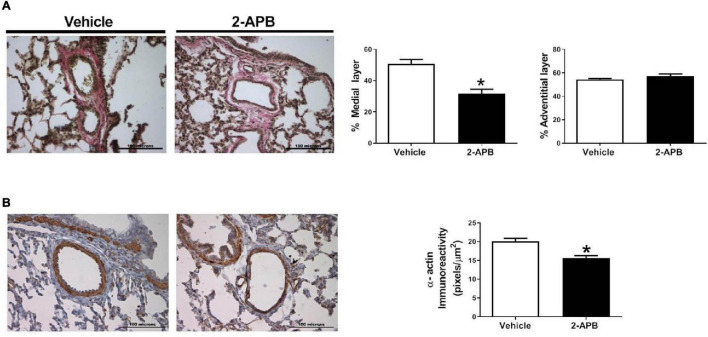
Small pulmonary arteries remodeling. Medial and adventitial layer area **(A)** and SM-α-actin **(B)** immunostaining of pulmonary arteries, performed in lung slices 48 h after the end of the treatment. Scale bar = 100 μm; magnification: 40×. Values are the mean ± SEM. *P ≤ 0.05, significant differences for vehicle vs. 2-APB, unpaired *t*-test.

The pulmonary vascular density was similar in both groups (Figures 5A,B), but the luminal vascular surface of the lung was increased in the 2-APB-treated lambs compared with the vehicle-treated lambs (Figures 5A,C). Pulmonary arteries from animals treated with 2-APB did not change the adventitial area but showed a decrease in the media layer area (Figure 6A) and in the immunoreactivity to SM-α-actin (Figure 6B) compared with the vehicle-treated lambs.

## Discussion

In this study, we tested whether treatment with 2-APB for 10 days can ameliorate the pulmonary arterial hypertension in hypoxic newborn lambs, conceived, gestated, born, and studied during their first 15 days of postnatal life at the Andean Altiplano at 3,600 m.a.s.l. This is a well-established model, showing increased basal mPAP and PVR, with also a greater increase in mPAP and PVR in response to acute hypoxia compared with the lowland controls (Herrera et al., 2007, 2019; Lopez et al., 2016; Gonzaléz-Candia et al., 2020). Our results showed that 2-APB-treated lambs had a lower mPAP between the first and the third day of treatment and during the fifth, the sixth, and the ninth day of treatment compared with the vehicle-treated lambs (*P* < 0.001 for the global effect of the factor treatment in two-way ANOVA), but at the end of the treatment, mPAP did not differ between groups. Nevertheless, the AUC for mPAP, as an integrated assessment of this variable along with the treatment, was lower in the 2-APB-treated group. The postnatal evolution of basal HR, CO, mSAP, and SVR was unaffected by the treatment, in agreement with previous findings on acute administration of a single dose of 2-APB (Parrau et al., 2013). The net basal PVR was similar in the vehicle- and 2-APB-treated high-altitude lambs at the end of the treatment, but in the latter, the AUC during the treatment was lower in agreement with analogous observation in mPAP. The increase in mPAP in response to acute hypoxic stress evaluated 24 h after the end of the treatment was slightly attenuated by the treatment, but neither PVR nor CO responses to hypoxia were modified despite a significant decrease in pulmonary vascular remodeling. In a previous study, our group observed that a single dose of 8 mg/kg of 2-APB did not modify the basal mPAP or PVR in either low- or high-altitude lambs, but it attenuated the hypoxic pulmonary vasoconstriction in both when measurements are carried out immediately after the drug infusion. Moreover, the inhibitory action of 2-APB in these responses was greater in the high-altitude lambs than in the low-altitude lambs (Parrau et al., 2013). The marked attenuation of hypoxic pulmonary vasoconstriction with a single dose of 2-APB observed in our previous study compared with its mild action observed with repetitive doses over 10 days may result from differences in the time spent between the drug infusion and the acute hypoxic challenge test. This is suggestive of rapid clearance of 2-APB away from the pulmonary circulation in high-altitude hypoxemic lambs. In our previous studies performed on highland lambs, we suggested that a similar single dose of this compound would allow reaching a concentration in the 10–100 μM range in the extracellular space, assuming a distribution volume of 40% of the body weight. The same concentration order blocks SOC in the pulmonary arteries isolated from those animals (Parrau et al., 2013) and in HEK293 cell lines transfected with Orai/Stim or TRPC subunits (Lievremont et al., 2005; DeHaven et al., 2008; Peinelt et al., 2008). Nevertheless, a pharmacokinetic study is needed to elucidate the time needed to reach maximal plasma and tissue concentration and the clearance rate of 2-APB. In fact, when measured *ex vivo*, under conditions of blockade of voltage-dependent Ca^2+^ channels with nifedipine, the pulmonary arterial contraction elicited by voltage-independent Ca^2+^ entry from extracellular space was upregulated in high-altitude lambs, and the relaxation in response to either 2-APB or SKF-96365, another non-selective TRPC channels inhibitor was also greater. These responses are in agreement with the upregulation of Stim1 and TRPC4 transcripts in lungs from high-altitude lambs (Parrau et al., 2013). Taken together, these findings, in addition to extensive pharmacological characterization of 2-APB on Ca^2+^ signaling, suggest that cardiopulmonary actions of this drug in newborn lambs are the result of their action on SOC, even though we cannot preclude its targeting on intracellular Ca^2+^ sources, such as IP_3_ receptors, as discussed below (reviewed in Putney, 2010; Parrau et al., 2013). 2-APB also inhibits TRPM7 channels and connexin proteins from GAP junctions. Nevertheless, it seems unlikely that cardiopulmonary actions observed here are related to these targets because neither TRPM7 nor 2-APB-sensitive connexins are upregulated in the pulmonary vasculature under chronic hypoxia, and the concentrations needed to inhibit the former are excessively high and related to acidification of intracellular milieu rather than direct action on the channel (Bai et al., 2006; Billaud et al., 2011; Chokshi et al., 2012). There is also evidence that 2-APB can act as a reactive oxygen species (ROS) scavenger, and in doing so, it protects cardiomyocytes, ovary, and testis tissue against ischemia/reperfusion damage (Taskin et al., 2014; Sari et al., 2015; Morihara et al., 2017). Inhibition of oxidative stress through melatonin administration increases NO bioavailability and NO-dependent vasodilation of pulmonary arteries in highland lambs (Torres et al., 2015). It is possible that 2-APB, under our experimental conditions, produces similar effects as melatonin, increasing NO bioavailability, but in that case, the NO-vasodilation would be limited by blunted sGC observed in highland lambs (Herrera et al., 2008a). Previously, we reported the effects of a similar treatment with 2-APB but in lambs with partial gestation and birth at high altitude, and postnatally raised at low altitude under normoxia (Castillo-Galán et al., 2016). These lambs have also increased PAP and resistance, enhanced pulmonary vasoconstrictor response to acute hypoxic pulmonary vasoconstriction and pulmonary arterial remodeling compared with the control lambs gestated, born, and studied at low altitude (Herrera et al., 2010). Nevertheless, these lambs are normoxemic, while the lambs used in this study are hypoxemic (Herrera et al., 2010, 2019). When the hemodynamic effects of the treatment with cumulative doses of 2-APB are compared in both models, we observed that basal mPAP decreases from ∼31 mmHg at the beginning of this study to ∼21 mmHg at the end, while the initial and final PVR were ∼0.071 and ∼0.058 mmHg ml kg^–1^ min^–1^, respectively. In contrast, in lambs with partial gestation at high-altitude basal mPAP decreases from ∼22 mmHg at the beginning of the treatment to ∼15 mmHg at the end, while basal PVR lowers from ∼ 0.06 to ∼0.045 mmHg ml kg^–1^ min^–1^ (Castillo-Galán et al., 2016). In contrast, under acute hypoxic challenge after the end of the treatment, mPAP and PVR reached ∼38–40 mmHg and 0.105 mmHg ml kg^–1^ min^–1^ in high-altitude lambs from this study. A similar response is reported in previous studies (Parrau et al., 2013; Lopez et al., 2016; Herrera et al., 2019). After 2-APB treatment, hypoxic mPAP and PVR were ∼35 mmHg and 0.081 mmHg ml kg^–1^ min^–1^, respectively. In lambs with partial gestational hypoxia, the mPAP and PVR under acute hypoxia reached ∼32 mmHg and ∼0.085 mmHg ml kg^–1^ min^–1^, respectively, while 2-APB treatment markedly lowered these values to ∼22 mmHg and ∼0.055 mmHg ml kg^–1^ min^–1^, respectively (Herrera et al., 2010; Castillo-Galán et al., 2016). We speculate that the higher initial mPAP of lambs used in this study, as well as their hypoxemic state, compared with lambs with partial gestation at high altitude but treated under normoxia could partially explain these differences. Both pulmonary vasoconstriction and remodeling induced by the environmental hypoxia are related to the enhancement of store-operated Ca^2+^ signaling through rapid association and upregulation of their Stim, Orai, and TRPC protein components, respectively (Lin et al., 2004; Ng et al., 2012; Hou et al., 2013; Chen et al., 2017; Wang et al., 2017; He et al., 2018; Reyes et al., 2018a). Increased basal mPAP probably results from the combination of hypoxic pulmonary vasoconstriction together with the pulmonary arterial remodeling in the hypoxemic lambs, while it could mainly result from remodeling in our previous study with normoxemic lambs with less severe disease (Castillo-Galán et al., 2016). Therefore, the administration of the combination of postnatal reoxygenation and 2-APB results in a better reversal of pulmonary arterial hypertension. It is interesting to note that neither CO nor SAP and resistance was modified by 2-APB, under basal conditions or acute hypoxic challenge, reinforcing the idea that vasoactive mechanisms modified by 2-APB are functionally present in the pulmonary vessels than in other vascular beds as described for SOC (Snetkov et al., 2003). When the action of 2-APB on pulmonary vascular remodeling in both ovine models is compared, similarities and differences are found. In this study, we detected a decrease in pulmonary arterial medial layer area in 2-APB-treated lambs, associated with increased luminal vascular surface and decreased α-actin immunoreactivity, while the adventitial layer was not modified. In contrast, α-actin staining, medial, and adventitial layers are diminished in normoxemic pulmonary hypertensive lambs treated with 2-APB (Castillo-Galán et al., 2016). The attenuated regression of remodeling involving only the medial layer in hypoxic pulmonary hypertensive lambs against medial and adventitial layers in normoxic pulmonary hypertensive lambs could be also a partial explanation of the difference in pulmonary hypertension improvement after 2-APB treatment in both models, but this assumption needs further investigation. Regardless of the model, basal mPAP does not normalize to the ∼10–12 mmHg and PVR does not reach the values of ∼0.04 mmHg ml kg^–1^ min^–1^ or lower reported for healthy lowland lambs (Herrera et al., 2007, 2010, 2019). Moreover, in this study, we observed a rapid initial decrease in basal mPAP at the beginning of the treatment followed by stabilization on the following days. Various explanations are possible for this finding. First, other sources of Ca^2+^ influx, such as L-type or T-type Ca^2+^ channels, could be present and compensate as SOCE inhibition by 2-APB is taking place. Both L- and T-type channels are upregulated in pulmonary arteries from hypoxic adult mice and contribute to pulmonary hypertension (Rodman et al., 2005; Wan et al., 2013; Chevalier et al., 2014). Whether those channels contribute to neonatal pulmonary hypertension in hypoxic lambs remains to be established. Second, the lack of selectivity of this drug and its action on other targets, such as IP3 receptors, could play a role. IP3 receptors contribute to Ca^2+^ release, Ca^2+^ store depletion, and SOCE activation in pulmonary arterial smooth muscle. However, it has been recently reported that the type II IP3 receptor subtype can also play a counter-regulatory role by limiting excessive intracellular Ca^2+^ increase through inhibition of SOCE, vasoconstriction, and remodeling (Shibata et al., 2019). At micromolar concentration, 2-APB could simultaneously block Stim1/Orai1 interaction, and IP3 receptor II, and also activates Orai3 channels in a Stim-independent way (Reyes et al., 2018a; Rosenberg et al., 2019). Consequently, cumulative treatment with 2-APB may prevent both Stim1-dependent activation of Orai1 and subsequent SOCE, but it also could attenuate the counter-regulation dependent on type II IP3 receptors. In addition, this can stimulate Stim1-independent Ca^2+^ entry through Orai3, limiting, in this way, the regression of pulmonary hypertension. The use of emerging drugs blocking selectively Orai1, independently of its interaction with Stim1 or IP3 receptors, such as AnCoA4 or CM-4620, are needed to validate SOCE as a pharmacological target against hypoxic pulmonary hypertension can achieve normalization (Reyes et al., 2018a; Stauderman, 2018; Waldron et al., 2019).

## Conclusion

The treatment of hypoxic pulmonary hypertensive lambs with 2-APB, a non-selective inhibitor of store-operated Ca^2+^ signaling, has a mild effect on pulmonary hypertension and PAP response to acute superimposed hypoxia, without any significant action on other systemic cardiovascular variables. Considering that 2-APB is not a specific blocker, new selective inhibitors for Orai1, the primary pore-forming subunit of store-operated Ca^2+^ channels, need to be assessed. The improvement promoted by 2-APB on hemodynamic variables in high-altitude hypoxemic newborn lambs is less marked than those previously documented by the same treatment on pulmonary hypertensive normoxemic lambs, despite significant regression of pulmonary vascular remodeling. Therefore, we suggest that oxygenation and 2-APB treatment could have additive actions in the reversal of pulmonary hypertension induced by perinatal hypoxia and validate this signaling pathway as a potential complementary treatment for hypoxic pulmonary arterial hypertension of the neonate.

## Data Availability Statement

The raw data supporting the conclusions of this article will be made available by the authors, without undue reservation.

## Ethics Statement

The animal study was all experimental protocols were reviewed and approved by the Faculty of Medicine Bioethics Committee of the University of Chile (CBA 0476 and 1172 FMUCH Acceptance Protocols).

## Author Contributions

SC-G, DP, SQ, FM, MD, GE, EH, IH, and AL performed the experiments. SC-G, SQ, GE, EH, RI, and AL analyzed the data. SC-G, SQ, EH, AL, RI, and RR interpreted the results of experiments. SC-G, DP, RR, AL, and GE prepared the figures. SC-G and RR drafted the manuscript. RR contributed to conception and design of research. All authors edited and revised the manuscript and approved final version of the manuscript.

## Conflict of Interest

The authors declare that the research was conducted in the absence of any commercial or financial relationships that could be construed as a potential conflict of interest.

## Publisher’s Note

All claims expressed in this article are solely those of the authors and do not necessarily represent those of their affiliated organizations, or those of the publisher, the editors and the reviewers. Any product that may be evaluated in this article, or claim that may be made by its manufacturer, is not guaranteed or endorsed by the publisher.
